# The Toxicity, Sublethal Effects, and Biochemical Mechanism of β-Asarone, a Potential Plant-Derived Insecticide, against *Bemisia tabaci*

**DOI:** 10.3390/ijms231810462

**Published:** 2022-09-09

**Authors:** Ran Wang, Yong Fang, Wunan Che, Qinghe Zhang, Jinda Wang, Chen Luo

**Affiliations:** 1 Institute of Plant Protection, Beijing Academy of Agriculture and Forestry Sciences, Beijing 100097, China; 2 Agriculture Biotechnology Institute, Hunan Academy of Agricultural Sciences, Changsha 410125, China; 3 Department of Pesticide Sciences, Shenyang Agricultural University, Shenyang 110866, China; 4 National Engineering Research Center of Sugarcane, Fujian Agricultural and Forestry University, Fuzhou 350002, China

**Keywords:** *Bemisia tabaci*, whitefly, β-asarone, plant-derived toxin, sublethal effects, metabolic enzymes, cytochrome P450 monooxygenases

## Abstract

*Bemisia tabaci* is a threat to agriculture worldwide because of its potential to cause devastating damage to various crops. β-asarone is a bioactive pesticidal chemical originating from *Acorus calamus* (or “Sweet Flag”) plants, and it displays significant lethal effects against insect pests. In this study, we established a baseline of susceptibility to β-asarone from China and patterns of cross-resistance to other popular insecticides. We found that all the 12 field-collected *B. tabaci* populations exhibited high susceptibility to β-asarone, and there was no cross-resistance detected for other tested insecticides. We subsequently evaluated the sublethal effects of β-asarone on physiology and biochemistry via LC_25_ treatment (4.7 mg/L). LC_25_ of β-asarone resulted in prolonged developmental duration and decreased survival rates in *B. tabaci* nymphs, pseudopupae, and adults. Significant reductions in oviposition duration, fecundity, and hatchability were also observed. Additionally, the metabolic enzyme activity and expression profiles of selected cytochrome P450 monooxygenase (P450) genes following the LC_25_ treatment of β-asarone suggest that enhanced detoxification via P450s could be involved in the observed sublethal effects. These findings demonstrate the strong toxicity and significant sublethal effects of β-asarone on *B. tabaci* and suggest that the induced overexpression of P450 genes could be associated with the response to β-asarone.

## 1. Introduction

*Bemisia tabaci*, the silverleaf whitefly, is a globally distributed, notorious insect pest for agricultural production, and it has been observed to cause significant damage to more than 600 species of plants, mainly by feeding on the phloem of host plants [1,2]. These insects can also damage plants indirectly by acting as vectors for over 100 plant viruses during the duration of feeding [3]. For more than 20 years, neonicotinoid insecticides have been the compounds of choice for controlling various arthropod pests due to their high toxicities [4], but there has been a recent spike in reports of field-evolved resistance to neonicotinoids in China [5]. Although whitefly populations have been effectively controlled in China by the use of chemical agents such as avermectins, novel neonicotinoids, anthranilic diamides, and butenolides for several years, a substantial reduction in the efficacy of the above chemical agents has been observed owing to the significant development of resistance to insecticides [5,6,7,8,9,10]. A widespread application of the chemical agents is, therefore, not a reasonable strategy when producing farm products with low residues of insecticides.

Plant-derived pesticidal chemicals have received much attention in recent years owing to their significantly high safety and low environmental impact. *Acorus calamus* (or Sweet Flag) is well-known in Asia for its pesticidal, antifungal, antibacterial, and medicinal properties [11,12,13]. Previous reports have demonstrated that the essential oil derived from the Sweet Flag plant show high lethal effects against *Lasioderma serricorne* and *Tribolium castaneum* and exhibit powerful repellent bioactive for *T. castaneum* [14]. Additionally, the use of the leaf-dip method with *A. calamus* essential oil has led to higher larval mortality in diamondback moths and tobacco cutworms [15]. *A. calamus* seems very promising for developing a safe plant-derived chemical agent for pest management, and it has been reported that β-asarone is the primary component isolated and purified from the essential oil of *A. calamus* and the main bioactive pesticidal chemical of *A. calamus*, which shows excellent toxicity to various insect pests [16,17]. In this context, unlike chemical insecticides, botanical pesticides composed of various bioactive chemicals provide the advantages of rapid degradation and the ability to act on the physiology and biochemistry of the targeted insect pests simultaneously [18,19,20]. They have been regarded as one of the practical and environmentally friendly strategies against agricultural insect pests [21].

Exposure to the sublethal concentrations of these compounds can cause a variety of physiological and behavioral alterations, negatively affecting the essential fitness components of arthropods such as prolonging the duration of growth and reducing the fecundity of F_1_ generation. The sublethal effects of insecticides on insect pests may be complex and diverse owing to differences in insecticide concentration/dose, insect species, treatment settings, or physiological conditions. Various classes of insecticides such as afidopyropen, clothianidin, cyantraniliprole, cycloxaprid, and dinotefuran have previously been shown to display sublethal effects on whiteflies [22,23,24,25,26]. There have also been reports of sublethal concentrations of β-asarone significantly decreasing longevity, fecundity, and egg hatchability in *Nilaparvata lugens* [27] insect populations, but the sublethal effects of β-asarone on *B. tabaci* have not been reported so far. In an effort to evaluate the latent capacity of β-asarone as a useful insecticide for populations of *B. tabaci*, we sought to confirm the compound’s sublethal effects on *B. tabaci*. More importantly, demonstration of the sublethal effects of this compound for a comprehensive evaluation of β-asarone could contribute to enhancing the efficacy against *B. tabaci*.

The lethal effects of β-asarone on adults of whitefly were determined, and twelve field-collected populations of *B. tabaci* collected from six provinces of China were monitored for their susceptibility to β-asarone. Researchers found that all of the 12 tested populations exhibited high susceptibility to β-asarone and minimal cross-resistance to other tested chemical agents. The sublethal effects of β-asarone on *B. tabaci* were then evaluated. Considering that cytochrome P450 monooxygenases (P450s), esterases (ESTs), and glutathione S-transferases (GSTs) in insects are related to tolerance and resistance to most popular chemical agents against *B. tabaci* [2,4,8,10], to illustrate the biochemical responses to the sublethal concentrations of β-asarone, the activities of three main detoxifying enzymes, namely P450s, ESTs and GSTs, were measured alongside the expression profiles of possibly related genes.

## 2. Results

### 2.1. Baseline Susceptibility to β-Asarone and Cross Resistance Pattern

On the basis of our previous work [10], the baseline susceptibility to β-asarone was established using twelve populations collected from six provinces of northern China in 2021 (Table S1). All the field populations were susceptible to β-asarone, with an LC_50_ that ranged from 11.78 to 42.80 mg/L (Table 1). Compared with the susceptible strain MED-S, the field populations showed no elevated resistance to β-asarone, as indicated by resistance ratios that varied from 0.8- to 2.8-fold. There was no evidence for cross-resistance to β-asarone in the flupyradifurone-resistant strain Flu-R (96.8-fold resistance to flupyradifurone), the cyantraniliprole-resistant strain CYAN-R (83.5-fold resistance to cyantraniliprole), and the afidopyropen-resistant strain HD-Afi (53.5-fold resistance to afidopyropen) (Table 2).

### 2.2. Sublethal Effects of β-Asarone on B. tabaci

**Figure 1:**
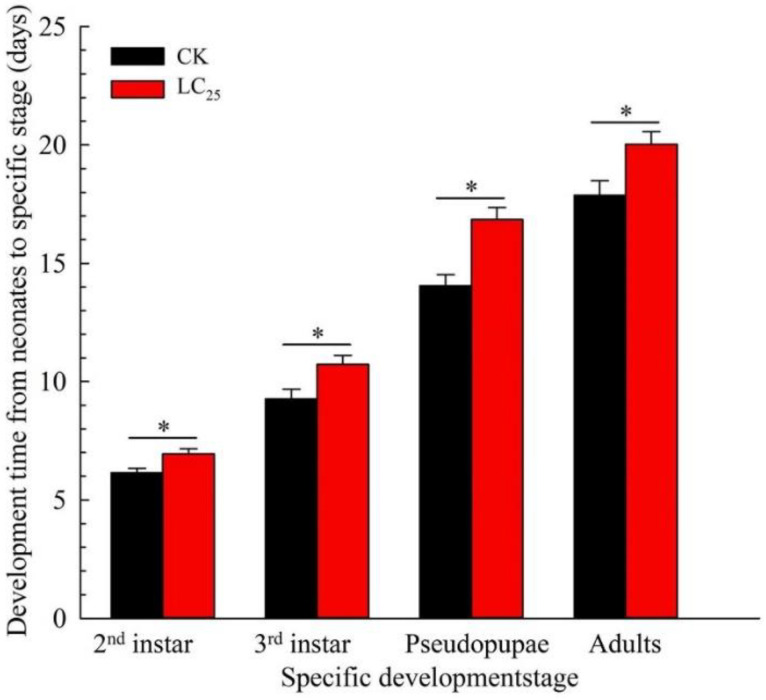
Development times for the F_1_ generations of *B. tabaci* in CK and LC_25_ treatment groups. Values are presented as means ± SE. Asterisks above error bars indicate significant differences (*p* < 0.05).

**Figure 2:**
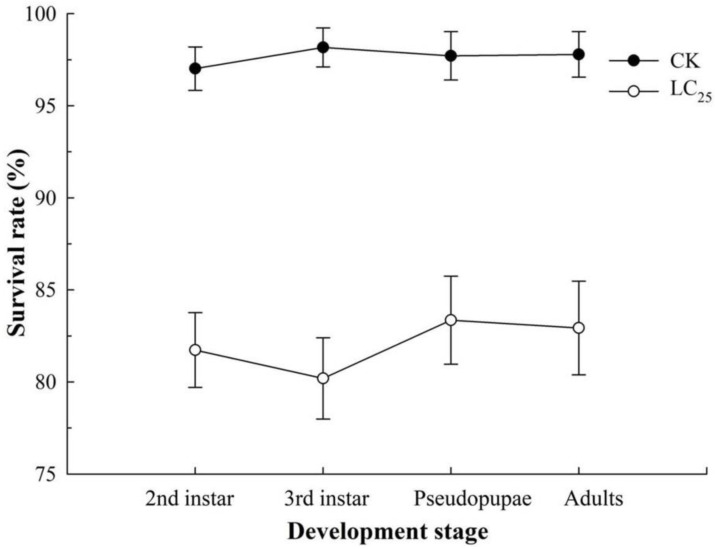
Survival rates of the F_1_ generations of *B. tabaci* in CK and LC_25_ treatment groups. Values are presented as means ± SE.

**Figure 3:**
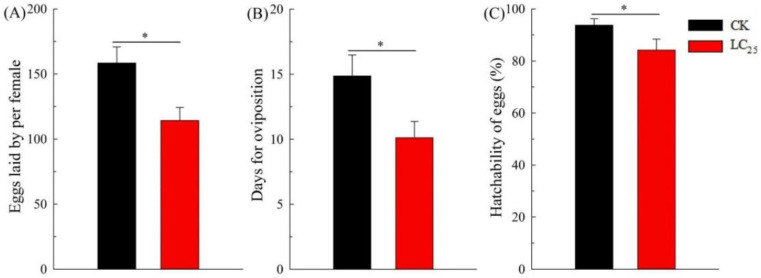
Fecundity (**A**), oviposition duration (**B**), and egg hatching rates (**C**) of the F_1_ generations of *B. tabaci* in CK and LC_25_ treatment groups. Values are presented as means ± SE. Asterisks above error bars indicate significant differences (*p* < 0.05).

We investigated the sublethal effects of β-asarone because insecticides are known to degrade under field conditions [28]. The LC_25_ of β-asarone on *B. tabaci* was measured at 4.7 mg/L. Exposure to LC_25_ of β-asarone extended the developmental time of each stage in comparison with the control ones (Figure 1). The survival percentage of the second instar, third instar, pseudopupae, and adults of *B. tabaci* significantly decreased in the group of LC_25_ treatment compared with the untreated group (Figure 2). Furthermore, exposure to LC_25_ treatment lowered the reproductive components including fecundity (114.28 ± 10.06 eggs/female) (Figure 3A) and oviposition duration (10.12 ± 1.25 days) (Figure 3B), compared with the control (158.44 ± 12.41 eggs/female and 14.87 ± 1.61 days, respectively). In addition, the hatchability of the LC_25_-treated group (84.19 ± 4.18%) (Figure 3C) was lower than that of the control (93.77 ± 2.46%).

### 2.3. Biochemical Mechanism of Response to LC_25_ of β-Asarone

**Figure 4:**
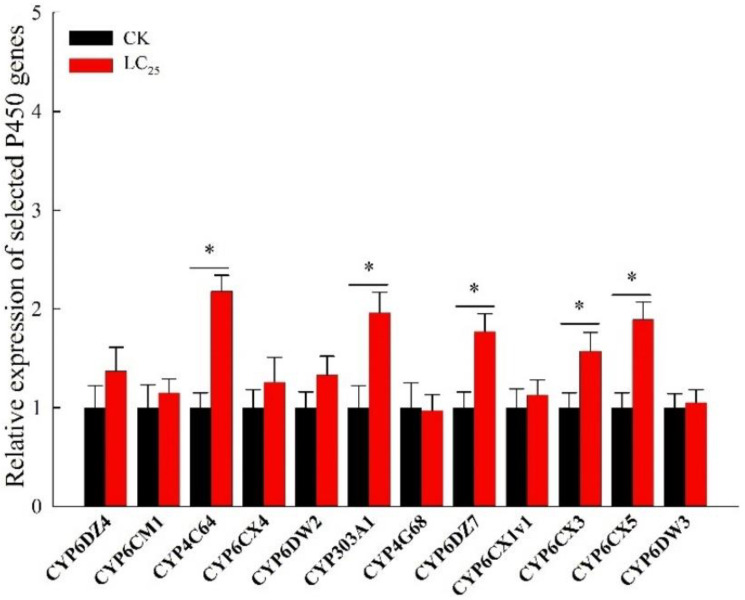
Expression profiles of 12 candidate P450 genes of the F_0_ generation of *B. tabaci* adults in the CK and LC_25_ treatment groups. Values are presented as means ± SE. Asterisks above error bars indicate significant differences (*p* < 0.05).

The activities of detoxification enzymes P450s, GSTs, and esterases were measured in both the CK and LC_25_ groups (Table 3). The activity of P450s in the LC_25_ group significantly increased 1.9-fold compared with that in the CK group. There was no significant difference between the activities of GSTs (elevated 1.3-fold) and esterases (elevated 1.1-fold). Of the twelve CYP candidates, five displayed significantly elevated gene expression in the sublethal treatment group compared with the control: *CYP4C64* (increased 2.2-fold), *CYP303A1* (increased 2.0-fold), *CYP6DZ7* (increased 1.8-fold), *CYP6CX3* (increased 1.6-fold), and *CYP6CX5* (increased 1.9-fold) (Figure 4)

## 3. Discussion

Plant-derived insecticides are receiving much attention due to their light impact on natural surroundings. Although botanical toxins are considered among the promising alternatives to the application of chemical agents, there are few known to control *B. tabaci* efficiently. β-asarone is the primary bioactive pesticidal chemical of *A. calamus* [16,29,30]. Against *N. lugens*, β-asarone achieves a 50% mortality at low concentrations as well as showing clear insecticidal activities against *Lasioderma serricorne* [12]. In the current work, we aimed to set the baseline susceptibility of β-asarone to *B. tabaci* adults from 12 field-collected populations from northern China. Furthermore, we investigated the potential patterns of cross-resistance to three popular chemical agents against *B. tabaci*. Our study indicated that β-asarone showed significant insecticidal activity with no cross-resistance to the three successfully commercialized chemical agents. The above results showed that β-asarone has a powerful lethal effect and offered a useful basis for further study and application of β-asarone against *B. tabaci*.

Although sublethal doses are not expected to directly kill insect pests, they have been reported to affect some of the most critical functions required for their survival [28]. Pests treated with the sublethal concentrations of insecticides exhibited decreases in fitness to varying degrees. Examples of the affected processes include a variety of stresses in behavioral processes, physiological activities, and overall general fitness [31]. Botanical pesticides not only have the potential capacity of insecticide but also affect the functions of insect physiology in a variety of ways including survival, development, reproduction, behavior, and metabolism [32,33]. In the present study, we found that the LC_25_ of β-asarone treatment prolonged the duration of growth and reduced the survival rates of whitefly nymphs, pseudopupae, and adults when compared with the control, and the periods of oviposition, fecundity, and the hatching rate of eggs were also significantly reduced. Similarly, the fecundity of females and the hatching rate of eggs were also significantly decreased in the *N. lugens* populations treated with sublethal concentrations of β-asarone [27]. The sublethal effects of chemical agents on arthropods may vary owing to differences in insecticide volume, treatment conditions, concentration, or species of arthropods. Thus, understanding the potential sublethal effects is essential when performing a comprehensive evaluation of an insecticide for use in an integrated pest management (IPM) program. In addition, the determination of the sublethal effects on insect pests contributes to enhancing the efficacy of control. Additionally, accurate assessments of the best control stage for β-asarone against insect pests, an improved understanding of the control effects of β-asarone, and a reduction in the costs of pest control are of particular importance.

When insect pests are treated with pesticidal chemicals, detoxifying enzymes are deployed in an effort to mitigate the effects. Former reports have shown that the P450 monooxygenases of *B. tabaci* play an essential role in the detoxification response to chemical pesticides [8,9,10,34]. Researchers found that the overexpression of P450 genes of the CYP4 and CYP6 clades in *B. tabaci* is involved in the processes of detoxification following exposure to various classes of chemical agents. It has been reported that two genes, CYP6CX4 and CYP4G68, are overexpressed in resistant strains of *B. tabaci* and have a confirmed association with resistance to flupyradifurone and cyantraniliprole [35,36]. In various field populations of *B. tabaci*, a series of P450 genes were shown to be associated with different levels of resistance to neonicotinoid insecticides [37,38]. Our analyses revealed that following treatment with β-asarone LC_25_, five P450 genes were significantly overexpressed in comparison to the control, suggesting that these P450 genes are involved in the processes triggered in response to β-asarone exposure. In the case of *N. lugens*, the involvement of several P450 genes in the detoxification metabolism of β-asarone has been confirmed [39,40]. We suspect that these P450 genes, some of which display significant overexpression, may encode for detoxifying enzymes, and that the lethal effects of β-asarone may be the result of the inhibition of these P450 genes, thus preventing effective detoxification in *B. tabaci*, resulting in their death. Although the possible mode of action of β-asarone on *B. tabaci* is not clear yet, the decreased longevity of adults and reduced fecundity display extra functions beyond lethal effects with the application of β-asarone against *B. tabaci*. Further studies in the lab will be performed to investigate the lethal and sublethal effects of β-asarone on the natural enemies of *B. tabaci*. Moreover, we plan to carry out RNA-seq and functional studies to study this mechanism further.

## 4. Materials and Methods

### 4.1. Insects

The *Bemisia tabaci* MED-S strain was originally collected from damaged poinsettia (*Euphorbia pulcherrima* Wild. ex Klotz.) in Beijing, China, in 2009 [41]. Three previously published resistant strains of whitefly were used to construct the cross-resistance pattern, including the flupyradifurone-resistant strain (FLU-SEL), the cyantraniliprole-resistant strain (CYAN-R), and the afidopyropen-resistant strain (HD-Afi) [34,35,42]. Twelve field populations of whiteflies were sampled across different regions of northern China [10], and all were determined to be the MED cryptic species [43]. All the tested populations of *B. tabaci* were reared on plants of cotton (*Gossypium hirsutum* L. var. “Shiyuan 321”) under the photoperiod of 16 L: 8D, the temperature of 26 ± 1 °C, and the relative humidity of 55 ± 5%. Adults of the tested *B. tabaci* no more than seven days old were sampled at random and utilized at about a 1:1 ratio of females and males for all the bioassays conducted for this study.

### 4.2. Insecticides and Chemicals

All the tested chemical agents were according to analytical standards. β-asarone (Sigma Aldrich, St. Louis, MO, USA, CAS# 5273-86-9, catalog# 02890590-25MG), flupyradifurone (Sigma Aldrich, CAS# 951659-40-8, catalog# 37050-100MG), cyantraniliprole (Sigma Aldrich, CAS# 736994-63-1, catalog#32372-25MG), and dimethyl sulfoxide (Sigma Aldrich, CAS# 67-68-5, catalog# D8418-500ML) were purchased from Sigma Aldrich in Shanghai, China. Afidopyropen (Dr. Ehrenstorfer, CAS# 915972-17-7, catalog# DRE-C10047000) was purchased from Dr. Ehrenstorfer in Augsburg, Germany.

### 4.3. Toxicity of β-Asarone on B. tabaci

To evaluate the susceptibility of *B. tabaci* strains to β-asarone and establish cross-resistance patterns with three other insecticides, bioassays were conducted on adults using an improved feeding method [44]. The technical compound of each insecticide was initially dissolved by dimethyl sulfoxide to 20,000 mg/L and then diluted with a diet solution into a series of dilutions for different working concentrations such as 80, 40, 20, 10, and 5 mg/L. Four replicates of at least five concentrations of each insecticide and the control group were conducted for each bioassay, and 30–40 adult whiteflies were collected at random for each one of the replicates. After 48 h, the number of surviving and dead adults of *B. tabaci* was counted, and the death rate of each treatment was calculated.

### 4.4. Sublethal Effects of β-Asarone on B. tabaci

On the basis of the bioassay conducted with five concentrations of β-asarone on the adults of *B. tabaci* shown above, the LC_25_ value was determined and utilized in the treatment of LC_25_ as a sublethal treatment strategy on *B. tabaci*, following the feeding steps shown above. Various fitness components were then recorded, including the duration of growth and the viability of developmental stages in the offspring, the duration of oviposition, the fecundity of females, and the hatching rate of their eggs. Fifty adults of *B. tabaci* were introduced into the tube with LC_25_ of β-asarone with two more tubes as replicas, and the tubes were placed in the chamber under the photoperiod of 16 L: 8D, the temperature of 26 ± 1 °C, and the relative humidity of 55 ± 5%. Additionally, another three tubes of *B. tabaci* adults with no exposure to LC_25_ of β-asarone were set as controls. After 48 h of feeding, the LC_25_ of β-asarone treatment and the control groups were ready to be transferred to the plants. Ten whitefly-free plants of cotton were moved into two individual insect-proof cages (one control cage for CK and one experimental cage for LC_25_ treatment) with five cotton plants in one of the cages. Plants of cotton in the LC_25_ cage were treated with β-asarone at the LC_25_ concentration, while the CK cage was without treatment and set as the group of controls. One hundred adults of *B. tabaci* that were treated (LC_25_) by the LC_25_ concentration were then moved into the LC_25_ cage for egg laying measurements. One hundred untreated *B. tabaci* adults were moved into the CK cage. After 24 h of laying eggs, all the plants were moved out of the two cages, and ten leaves were sampled at random from each of the cages. In each of the 10 leaves, 20 eggs were left on each leaf and kept with one leaf clip-cage. All the spots of the eggs on the tested leaves were labeled on the surface with one marker pen, and the cages were under the photoperiod of 16 L: 8D, the temperature of 26 ± 1 °C, and the relative humidity of 55 ± 5%. Newly emerged *B. tabaci* adults were then moved onto fresh leaves of cotton with clip cages for fecundity measurements that continued until all individuals died, and then the hatchability of eggs was counted and calculated.

### 4.5. Metabolic Enzyme Assays and Expression Patterns of Detoxification Related to P450s

Activities of P450 monooxygenases (P450s), esterases, and glutathione S-transferases (GSTs) were measured based on our previously reported method, with minor modifications [35]. Each strain was assayed in triplicate using 200 adults per replicate. Protein content was quantified using BSA as a standard according to Bradford’s method [45]. According to the previously published articles concerning P450-gene-associated insecticide resistance in *B. tabaci*, 12 candidate P450s, namely *CYP6CM1*, *CYP6DZ4*, *CYP4G68*, *CYP6DW2*, *CYP6CX4*, *CYP4C64*, *CYP303A1*, *CYP6DZ7*, *CYP6CX3*, *CYP6CX1v1*, *CYP6DW3*, and *CYP6CX5,* were chosen, and the gene expressions of the 12 candidate P450s were determined [37,38]. After 48 h treatment of β-asarone LC_25_, the total RNA was extracted from 100 *B. tabaci* adults randomly sampled from the treatment population. One hundred of the untreated *B. tabaci* adults were also randomly sampled and set as the CK group. The gene expression was measured via qPCR using *TUB1α* and *EF-1α* for normalizing the data. All the information on primer sequences is displayed in Table S2.

### 4.6. Statistical Analysis

Differences in mortality between various working concentrations were recorded, an analysis of the bioassay data collected at different working concentrations of the β-asarone was conducted, and LC_50_ values were evaluated using the PoloPlus software (LeOra Software, Berkeley, CA, USA, 2002) [46]. Treatment differences in the developmental time, survival rates, oviposition period, fecundity, and hatchability of *B. tabaci* treatments were determined via Student’s *t*-test. The metabolic activity and expression patterns of P450 genes were compared between the two treatment groups using Student’s *t*-test. All statistical analyses were implemented in the SPSS software (SPSS Inc., Chicago, IL, USA, 2011) [47].

## Figures and Tables

**Table 1:** Susceptibility to β-asarone in field-collected *B. tabaci* populations from China.

Population	N ^a^	LC_50_ (95% CL) (mg L^−1^) ^b^	Slope ± SE	*χ*^2^ (df)	RR ^c^
MED-S	732	15.39 (12.82–18.23)	1.31 ± 0.12	1.78 (3)	
LY	727	13.19 (10.50–16.34)	1.02 ± 0.12	1.43 (3)	0.9
CY	730	17.14 (13.69–23.04)	1.24 ± 0.13	2.35 (3)	1.1
HD	723	24.36 (20.40–29.06)	1.27 ± 0.12	2.07 (3)	1.6
TZ	736	31.90 (26.25–37.87)	1.32 ± 0.12	1.40 (3)	2.1
WQ	729	22.60 (18.52–27.39)	1.14 ± 0.12	1.26 (3)	1.5
JH	739	35.77 (30.20–42.17)	1.35 ± 0.12	1.90 (3)	2.3
ZJK	721	29.78 (25.19–34.43)	1.77 ± 0.14	1.64 (3)	1.9
BD	735	11.78 (9.62–14.36)	1.11 ± 0.12	2.33 (3)	0.8
ZZ	718	26.10 (21.42–30.88)	1.44 ± 0.13	1.82 (3)	1.7
XZ	731	42.80 (34.52–56.23)	1.14 ± 0.12	2.89 (3)	2.8
JN	727	16.06 (13.14–20.34)	1.12 ± 0.12	2.58 (3)	1.0
TA	734	21.61 (17.00–29.59)	1.01 ± 0.12	0.51 (3)	1.4

^a^ Number of insects used. ^b^ CL = confidence limits. ^c^ RR (resistance ratio) = LC_50_ (field-collected population)/LC_50_ (MED-S).

**Table 2:** Cross-resistance patterns to β-asarone and three popular insecticides against *B. tabaci*.

Insecticide	Strain	N ^a^	LC_50_ (95% CL) (mg/L) ^b^	Slope ± SE	*χ*^2^ (df)	RR ^c^
β-Asarone	MED-S	721	15.51 (13.71–17.47)	2.02 ± 0.14	2.32 (3)	
	HD-Afi	732	12.56 (9.97–15.13)	1.39 ± 0.13	1.64 (3)	0.8
	CYAN-R	722	14.33 (12.29–16.46)	1.73 ± 0.14	1.92 (3)	0.9
	FLU-SEL	724	16.74 (13.74–19.72)	1.62 ± 0.14	2.66 (3)	1.1
Afidopyropen	MED-S	725	13.13 (10.73–16.11)	1.10 ± 0.12	1.06 (3)	
	HD-Afi	737	702.38 (595.07–835.88)	1.33 ± 0.12	0.75 (3)	53.5
	CYAN-R	711	15.06 (12.04–18.22)	1.21 ± 0.12	2.77 (3)	1.1
	FLU-SEL	714	12.70 (9.58–15.87)	1.07 ± 0.12	2.27 (3)	1.0
Cyantraniliprole	MED-S	715	1.57 (1.32–1.88)	1.29 ± 0.12	1.31 (3)	
	HD-Afi	721	1.85 (1.55–2.15)	1.72 ± 0.14	2.45 (3)	1.2
	CYAN-R	719	131.08 (107.30–156.33)	1.29 ± 0.12	2.86 (3)	83.5
	FLU-SEL	706	1.28 (1.06–1.50)	1.60 ± 0.13	0.85 (3)	0.8
Flupyradifurone	MED-S	712	15.93 (12.65–19.40)	1.15 ± 0.12	1.25 (3)	
	HD-Afi	726	14.61 (12.09–17.36)	1.30 ± 0.12	2.49 (3)	0.9
	CYAN-R	711	12.44 (9.91–15.11)	1.17 ± 0.12	2.05 (3)	0.8
	FLU-SEL	721	1541.67 (1286.02–1895.20)	1.25 ± 0.12	1.71 (3)	96.8

^a^ Number of insects used. ^b^ CL = confidence limits. ^c^ RR (resistance ratio) = LC_50_ (HD-Afi or CYAN-R or FLU-SEL)/LC_50_ (MED-S).

**Table 3:** Activities of metabolic enzyme in populations with CK and LC_25_ treatment of *B. tabaci* ^a^.

Treatment	P450 Activity		EST Activity		GST Activity	
	pmol min^−1^ mg^−1^	Ratio ^b^	nmol min^−1^ mg^−1^	Ratio ^b^	nmol min^−1^ mg^−1^	Ratio ^b^
CK	0.68 ± 0.16		311.06 ± 18.93		40.26 ± 10.31	
LC_25_	1. 32 ± 0.24 *	1.9	329.75 ± 25.32	1.1	51.07 ± 11.12	1.3

^a^ Mean activity values in the same column followed by asterisks are significantly different (*p* < 0.05). ^b^ Ratio = activity of LC_25_ treatment/activity of CK.

## Data Availability

All relevant data are available from the corresponding author on request (wangran@ipepbaafs.cn).

## Supplementary Materials

The following supporting information can be downloaded at: https://www.mdpi.com/article/10.3390/ijms231810462/s1.
