# Supplementary material for: The Toxicity, Sublethal Effects, and Biochemical Mechanism of β-Asarone, a Potential Plant-Derived Insecticide, against Bemisia tabaci
Source: Int J Mol Sci. 2022 Sep 9;23(18):10462. doi: 10.3390/ijms231810462 (PMC9501876; doi:10.3390/ijms231810462)
Supplement: Supplementary file 1 [file ijms-23-10462-s001.zip › ijms-1900182-supplementary.pdf]

Table S1. Information of field-collected *Bemisia tabaci* samples from northern China.

Names of populations	Locations of collection	Sites of collection	Date	Host plant
LY	Liaoyang, Liaoning	41.19N, 123.11E	Aug 2021	Eggplant
CY	Chaoyang, Liaoning	41.59N, 120.50E	Aug 2021	Cucumber
HD	Haidian, Beijing	39.97N, 116.31E	Apr 2021	Tomato
TZ	Tongzhou, Beijing	39.73N, 116.69E	Jun 2021	Tomato
WQ	Wuqing, Tianjin	39.35N, 117.10E	Jun 2021	Tomato
JH	Jinghai, Tianjin	38.90N, 116.94E	Jun 2021	Tomato
ZJK	Zhangjiakou, Hebei	40.58N, 115.00E	July 2021	Pepper
BD	Baoding, Hebei	38.82N, 115.39E	July 2021	Tomato
ZZ	Zhengzhou, Henan	34.91N, 113.56E	July 2021	Cucumber
XZ	Xinzheng, Henan	34.33N, 113.75E	July 2021	Pepper
JN	Jinan, Shandong	36.78N, 117.23E	Aug 2021	Tomato
TA	Taian, Shandong	36.14N, 117.22E	Aug 2021	Tomato

Table S2. Primers used in quantitative real-time PCR.

Primer name	Sequence (5′–3′)
CYP6DZ4	Forward: CGTCAAGCACCGAGAATCAA
	Reverse: ATCCTTAGCGTGACCATCCTG
CYP6CM1	Forward: CACTCTTTTGGATTTACTGCACCC
	Reverse: GTGAAGCTGCCTCTTTAATGGC
CYP4G68	Forward: GGTGTATCATGGAGACTCT
	Reverse: GCTGGACTTCTTGTTGTAG
CYP6CX4	Forward: CGGTTACACGCTTCACACTTG
	Reverse: TGGGTTCAGGGTAAACGCTTTC
CYP6DW2	Forward: CGCTGGAAAAACATCCGCAC
	Reverse: TTTGCGTCCAGGTATCCGTT
CYP303A1	Forward: CGCTCCGGTACAATGCTAATCG
	Reverse: CCCATACACCTGTGTTTACCGAAT
CYP4C64	Forward: CCCTCAAACGGTCCTTCCAAC
	Reverse: GTAATTCTGCGTCTTCGTCAACTG
CYP6DZ7	Forward: CGGAACACGCCTGACGAAAG
	Reverse: GTAACGCACATTCTTCCATCTCTG
CYP6CX1v1	Forward: GTATCGGATTACGCCCTTCACC
	Reverse: GCAGCCAAACTTCACCTTTCG
CYP6CX3	Forward: CGCATTCTTCCAGTTCCTCGAGA
	Reverse: GGCCATAGCATCCTTCGTGACC
CYP6CX5	Forward: GACTTTCCAGCTGCTCAACCC
	Reverse: GTTCCCGCTGAGCTTGTCCA
CYP6DW3	Forward: CTTACGAATTACCGAACTCAC
	Reverse: CGAACTTCTCAGGCTTAGG
TUB1α	Forward: CACTGTTGTTCCTGGTGGC
	Reverse: AGTGGACGAAAGCACGCTTG
EF1α	Forward: TAGCCTTCGTGCCAATTTCCG
	Reverse: CCTTCAGCATTACCGTCC
